# Real Life Multicenter Comparison of 24-Month Outcomes of Anti-VEGF Therapy in Diabetic Macular Edema in Turkey: Ranibizumab vs. Aflibercept vs. Ranibizumab-Aflibercept Switch

**DOI:** 10.3390/medicina59020263

**Published:** 2023-01-30

**Authors:** Murat Kucukevcilioglu, Yağmur Seda Yeşiltaş, Ali Hakan Durukan, Nurten Unlu, Mehmet Onen, Mehmet Numan Alp, Defne Kalayci, Mehmet Akif Acar, Mehmet Ali Sekeroglu, Mehmet Citirik, Ayse Gul Kocak Altintas, Dicle Hazirolan, Pinar Cakar Ozdal, Yasin Toklu, Tolga Bicer, Nagihan Ugurlu, Ozlem Budakoglu, Zeliha Yazar, Nil Irem Ucgun Zeki, Kurtulus Serdar, Sibel Doguizi, Yasemin Ozdamar Erol, Cemile Ucgul Atilgan, Mucella Arikan Yorgun, Dilek Ozcelik Soba, Nilufer Berker, Ceyda Baskan

**Affiliations:** 1 Department of Ophthalmology, Gulhane School of Medicine, University of Health Sciences, 06010 Ankara, Türkiye; 2 Department of Ophthalmology, Ankara Training and Research Hospital, 06230 Ankara, Türkiye; 3 Department of Ophthalmology, Ankara City Hospital, 06800 Ankara, Türkiye; 4 Department of Ophthalmology, Ankara Ulucanlar Training and Research Hospital, 27300 Ankara, Türkiye; 5 Department of Ophthalmology, Dışkapı Yıldırım Beyazıt Training and Research Hospital, 06110 Ankara, Türkiye; 6 Augenland MVZ Goethaplatz, 60311 Frankfurt, Germany

**Keywords:** anti-vascular endothelial growth factor (anti-VEGF), diabetic macular edema, macular thickness, real-world, switch, visual acuity

## Abstract

The aim of this study was to compare the outcomes of diabetic macular edema (DME) treated with aflibercept (AFB) or ranibizumab (RNB) only, and after switching from RNB to AFB. This was a retrospective, real-world, multicenter (7 cities) 24 month study. Overall, 212 eyes in the AFB group, 461 in the RNB group, and 141 in the RNB to AFB group were included. The primary endpoints were differences in visual acuity (VA) and central macular thickness (CMT) from baseline to the final visit. The secondary outcomes were the percentage of eyes that achieved ≥10 letters gain and ≥10 letters loss in vision at month 12 and 24, and the percentage of eyes that achieved a thinning of ≥20% in CMT at month 3 and month 6. The results showed that VA did not significantly differ at baseline (AFB: 0.62 ± 0.38, RNB: 0.61 ± 0.36, RNB to AFB: 0.61 ± 0.38), at checkpoints, or at the final visit (AFB: 0.46 ± 0.38, RNB: 0.5 ± 0.37, RNB to AFB: 0.53 ± 0.36) (*p* > 0.05). Though the mean CMT at baseline was significantly thicker in the RNB to AFB group (479 ± 129.6 μm) when compared to the AFB (450.5 ± 122.6 μm) and RNB (442 ± 116 μm) groups (*p* < 0.01), similar measurements were obtained after 12 months. The percentages of eyes that gained or lost ≥10 letters in the AFB, RNB, and RNB to AFB groups at year 1 and 2 were similar, as was the percentages of eyes that demonstrated ≥20% CMT thinning at month 3 and 6. Our study showed similar visual improvements in non-switchers (AFB and RNB groups) and switchers (RNB to AFB group) through 2 years follow-up, however, AFB patients required fewer injections, visits, or need for additional treatments.

## 1. Introduction

The incidence of diabetes mellitus (DM) is on the rise, as the 2021 International Diabetes Federation report revealed. Their report, consisting of data from 215 countries, revealed that the number of patients increased 3.5 fold (151 million to 537 million) since 2000, and is expected to be nearly 783 million in 2045 [1]. A study investigating the global prevalence (from 2015 to 2019) of diabetic retinopathy (DR) evaluated 32 papers from 21 different countries and found numbers of 27% for DR and 4.6% for diabetic macular edema (DME), which is the leading cause of vision loss [2]. Visual loss is the most feared complication among newly diagnosed DM patients [3]. Anti-vascular endothelial growth factor (VEGF) agents are recommended as first-line therapy for DME, since VEGF has been identified as one of the major factors contributing to the blood–retinal barrier breakdown. The efficacy and safety of anti-VEGF drugs in patients with DME have already been proven in landmark randomized controlled trials (RCTs) [4,5,6,7]. A head-to-head comparison trial protocol T study found that aflibercept (AFB) (2 mg) was more effective than ranibizumab (RNB) (0.3 mg) in improving vision at 1 year in eyes with very low visual acuity (VA) at the presentation of ≤68 letters (Snellen equivalent 20/50), while there was no difference in those presenting with VA ≥69 letters (Snellen equivalent 20/40), but the superiority of AFB over RNB, noted at 1 year, was no longer identified at 2 years [8,9]. Evidence from RCTs may not apply to real-world practice, where people in need of antiangiogenic treatment are often under-treated and under-monitored. Therefore, population-based post-marketing observational studies are required to see if these results of clinical trials are replicable in the general population.

Despite the clinical effectiveness of the currently used anti-VEGF agents, some patients show persistence of DME reaching a rate of 30–65% in clinical trials [10,11]. When DME does not respond enough after 3 to 6 injections, many ophthalmologists switch to another anti-VEGF, especially if the initial agent is bevacizumab (BVZ) [12,13]. A meta-analysis showed patients with persistent DME may obtain significant visual and anatomical improvement up to 12 months after switching to AFB [14]. However, other pivotal clinical trials, such as RISE/RIDE, and DRCR.net studies have demonstrated that these patients may achieve further functional and anatomical improvement with sustained treatment, which is called “late respond” [15,16,17]. Similarly, two other single center studies found no difference in visual outcomes between switched and non-switched eyes [18,19]. When all these data are considered, it is hard to decide whether this clinical improvement originates from the new anti-VEGF agent or continued treatment with anti-VEGF injections.

In light of the above, the purpose of this comparative, retrospective, multicenter study was to compare anatomical and visual outcomes (at 12 and 24 months) in DME patients treated with the same anti-VEGF agents (AFB or RNB) and in patients who switched from RNB to AFB.

## 2. Materials and Methods

### 2.1. Study Design

This was a retrospective, observational, multicenter study of consecutive patients with center-involving DME treated with intravitreal anti-VEGF injections (RNB and AFB) between April 2007 and February 2017. The Turkish Social Security Institution issued a new ruling in 2017, stating that three doses of BVZ should be administered first in patients who require anti-VEGF treatment, with additional anti-VEGF medicines being used only in resistant and/or unresponsive individuals. Therefore, we did not include patients with DME who were treated after 2017 in this study. In total, 26 ophthalmologists from 7 ophthalmology centers in Turkey participated in this study. Patients were treated with intravitreal anti-VEGF injections based on the routine practice of the ophthalmologist. Diabetic eyes with evidence of residual intraretinal and/or subretinal fluid, and central macular thickness (CMT) on optical coherence tomography (OCT) > 300 µm after receiving at least three RNB injections were considered to have persistent DME and could be switched to AFB. Additional adjunctive treatments could be given at any time point, according to the ophthalmologist’s discretion. This study was conducted in compliance with the tenets of the Declaration of Helsinki. The study was approved by the Research Ethics Committee of Gülhane Training and Research Hospital.

### 2.2. Study Population

The medical records of patients who were either treatment naïve or previously treated with a diagnosis of center-involving DME with CMT of >300 µm were reviewed. Inclusion criteria were: (1) age 18 years or older, (2) best corrected visual acuity (BCVA) of ≥20 letters (Snellen equivalent 20/400) on an Early Treatment Diabetic Retinopathy Study (ETDRS) chart, and (3) having a medical record of follow-up of at least 24 months. Exclusion criteria were: (1) secondary macular edema from other retinal diseases, (2) any concomitant ocular disease that could compromise VA, (3) a history of vitreoretinal surgery, (4) intravitreal anti-VEGF and/or steroid injection within three months prior to study inclusion, and (5) macular laser photocoagulation within three months before inclusion. If both eyes of a patient were eligible, both were included in the analysis.

### 2.3. Data Collection

The medical data of patients at baseline and every visit (every 3 months) up to month 24 were collected. Demographics and clinical characteristics, including type, duration, and stage of diabetes mellitus, ocular history, prior treatments, and other systemic medical diseases, were noted at baseline. At each subsequent visit, BCVA (ETDRS letters), CMT, intravitreal anti-VEGF injections, concomitant treatments for DME, and systemic or ocular adverse events (AEs) related to intravitreal anti-VEGF treatment were recorded. Snellen VA values were converted to approximate ETDRS line scores for analysis and calculated as follows: 85 + 50 × log (Snellen fraction) [20]. OCT scans were obtained by Spectralis OCT (Heidelberg Engineering, Heidelberg, Germany) or Cirrus OCT (Zeiss, Dublin, CA, USA) depending on the device available at each ophthalmology center. The CMT values automatically measured by the incorporated machine software were recorded. Cirrus OCT measurements were converted by an equation (Spectralis = 40.78 + 0.95 × Cirrus) as recommended by DRCR.net. [21].

### 2.4. Endpoints

The patient eyes were divided into three cohorts: (1) aflibercept injected only (AFB), (2) ranibizumab injected only (RNB), and (3) eyes that were switched from ranibizumab to aflibercept (RNB to AFB). The primary endpoints were changes in BCVA and CMT from baseline to final visit in each cohort, and the frequency of visits and intravitreal anti-VEGF injections. The secondary outcomes were the percentage of eyes that achieved a greater than 10 letters gain and more than 10 letters loss in vision at month 12 and 24. Additionally, we investigated the percentage of eyes that achieved a thinning of ≥20% in CMT (called “good responders”) at month 3 and month 6, as the treatment switch between anti-VEGFs is mostly performed at these time intervals.

### 2.5. Statistical Analysis

Data was recorded with the Microsoft Excel 2010 program and added to IBM SPSS Statistics 25 for analysis. The Shapiro–Wilk and the Kolmogorov–Smirnov tests were employed to check normality. Results are expressed as mean and standard deviation (SD) if the variables are continuous, and as frequencies and percentages if the variables are categorical. Baseline variables were compared between treatment groups using the one-way ANOVA test in the continuous variables and with the Chi-squared and Fisher tests for categorical variables.

## 3. Results

Table 1 shows the demographic and clinical characteristics of our study sample. A total of 814 eyes (212 AFB, 461, RNB, and 141 RNB to AFB) from 684 patients were included for analysis. The mean age of patients did not differ among three groups (*p* = 0.74). Nearly half of the patients were male or female, except for in the RNB to AFB group in which almost two thirds were male (*p* = 0.002). There was no difference in the type of diabetes among study groups (*p* = 0.074). The mean duration of DM was nearly 14 years among study groups (*p* = 0.396), and the distribution of diabetes treatment (insulin alone, oral antidiabetic alone, or insulin + oral antidiabetic) was similar (*p* = 0.349). Mostly, included eyes were right eyes (AFB = 51.6%; RNB = 53%; RNB to AFB = 54.4%), and treatment naïve (AFB = 62.7%; RNB = 62.1%; RNB to AFB = 56.4%; *p* = 0.4). There were no statistically significant differences among study groups regarding prior DME treatment (*p* = 0.331), lens status (mostly phakic, *p* = 0.063), DR grade (mostly non-proliferative DR, *p* = 0.339), and presence of systemic comorbidities (*p* = 0.02). AFB treated eyes had more panretinal laser photocoagulation before the study (AFB = 33.4%; RNB = 26.6%; RNB to AFB = 29.5%; *p* < 0.01).

Table 2 shows the clinical characteristics of study groups during the treatment period. The 3 loading dose treatment was completed significantly more in the RNB to AFB group (63.8%) when compared to AFB (55.8%) and RNB (54%) groups (*p* < 0.01). However, there was no significant difference in loading dose completion time among groups (*p* = 0.091). The mean number of injections before RNB to AFB switch was 5.82 ± 2.2 (range: 3–10). The mean number of visits in the first year was similar (around 7 visits) in all groups (*p* = 0.55). However, the AFB group showed significantly more visits (4.12 ± 1.9) in the second year when compared to the RNB (6.28 ± 2.1) and RNB to AFB (6.8 ± 2.1) groups (*p* < 0.01). In the first year, eyes in AFB group received significantly fewer injections (4.43 ± 1.7) in comparison to the RNB (5.3 ± 1.8) and RNB to AFB (5.39 ± 1.8) groups (*p* < 0.01). However, when it comes to the second year, eyes in the RNB to AFB group received significantly more injections (3.48 ± 1.9) when compared to the AFB (1.92 ± 1.5) and RNB (1.98 ± 1.8) groups (*p* < 0.01).

The most frequent additional treatments for macular edema among study groups were intravitreal dexamethasone implant injection (AFB = 11.3%; RNB = 15.7%; RNB to AFB = 17.4%) and focal/grid/subthreshold laser photocoagulation (AFB = 17.4%; RNB = 25.3%; RNB to AFB = 32.1%). A summary of ocular and systemic AEs is also presented in Table 2. The most frequent ocular AE was an increase in intraocular pressure (AFB = 2; RNB = 5; RNB to AFB = 1). The frequency of systemic embolic events was found to be very low among treatment groups.

Table 3 shows VA and CMT changes among study groups. The mean BCVA at baseline was 0.62 ± 0.38 logMAR in the AFB group, 0.61 ± 0.36 in the RNB group, and 0.61 ± 0.38 in the RNB to AFB group, showing no difference among study groups (*p* = 0.83). The mean BCVA did not differ among study groups at any 3 month interval during the 24-month follow-up, except for at month 6, which showed slightly significant better mean BCVA in the AFB group (0.44 ± 0.34) in comparison to the RNB (0.5 ± 0.38) and RNB to AFB groups (0.5 ± 0.37) (*p* = 0.049). The mean CMT at baseline was significantly thicker in the RNB to AFB group (479 ± 129.6 μm) when compared to the AFB (450.5 ± 122.6 μm) and RNB groups (442 ± 116 μm) (*p* < 0.01). This difference was maintained with significantly thicker CMT values at each point up to 12 months, including the 12th month CMT values (AFB: 333 ± 95.2 μm, RNB: 342 ± 119.1 μm, RNB to AFB: 369.2 ± 123.7 μm) (*p* < 0.01 for all). However, there was no significant difference at each point in the 2nd year up to 24 months, including the 24th month values (AFB: 366.78 ± 112.1 μm, RNB: 369.8 ± 104.6 μm, RNB to AFB: 373.4 ± 163.1 μm) (*p* > 0.05 for all).

**Figure 1:**
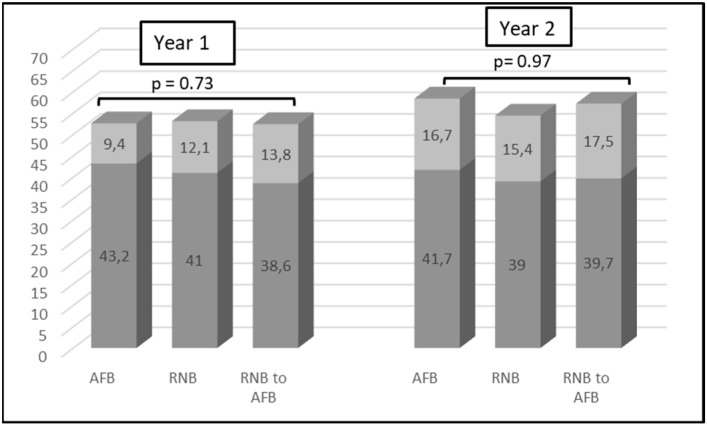
The percentages of eyes that achieved more than 10 letters gain and more than 10 letters loss in vision at month 12 and 24 among study groups.

Figure 1 shows the percentage of eyes that achieved more than 10 letters gain and more than 10 letters loss in vision at month 12 and 24 among study groups. At year 1, the percentage of eyes that achieved more than 10 letters gain from baseline VA was 43.2%, 41%, and 38.6%, and the percentage of eyes that lost more than 10 letters were 9.4%, 12.1%, and 13.8% in the AFB, RNB, and RNB to AFB groups, respectively. At year 2, the figures were 41.7%, 39%, and 39.7% for vision gainers, and 16.7%, 15.4%, and 17.5% for vision losers in groups AFB, RNB, and RNB to AFB, respectively. There were no significant differences between all groups in the change of vision at year 1 and year 2 when compared to baseline (*p* = 0.73 and *p* = 0.97, respectively).

**Figure 2:**
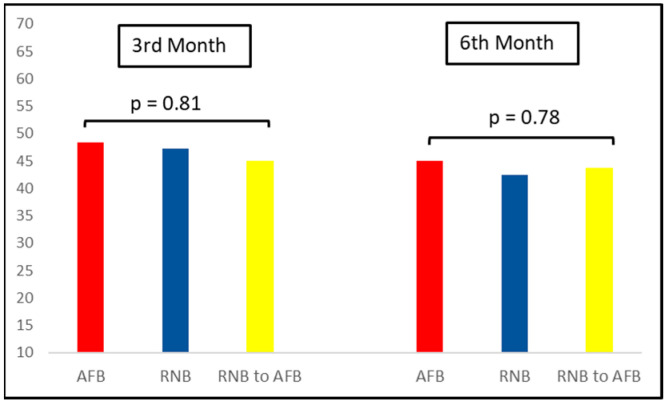
The percentages of eyes that demonstrated a decrease in central macular thickness (CMT) ≥20% at month 3 and month 6 among study groups.

Figure 2 shows the percentage of eyes that demonstrated a decrease in CMT ≥ 20% at month 3 and month 6. At month 3, the percentages were 45%, 43.7%, and 42.4% in groups AFB, RNB, and RNB to AFB, respectively. The percentages for month 6 were 48.4%, 47.3%, and 45% in groups AFB, RNB, and RNB to AFB, respectively. There were no significant differences between all groups at month 3 and month 6 (*p* = 0.81 and *p* = 0.78, respectively).

## 4. Discussion

Dugel et al., in a sub-analysis of Protocol I, investigated the relationship between early OCT response and long-term visual outcomes [22]. After sensitivity analyses, they determined a cutoff value (CMT change of <20% and ≥20%) to design study groups. We used the same cutoff value to explore the differences in early anatomic response across study groups at month 3 and 6. We noted similar proportions of eyes showed CMT reduction of ≥20% in the AFB (45%), RNB (43.7%), and RNB to AFB (42.4%) groups at month 3. With sustained treatment, we saw further improvement in CMT from month 3 to month 6, just as Dugel et al. reported. However, this improvement was not as good as Dugel et al.’s, as they reported a rate of 64.8% for the RNB group at 12 weeks in Protocol I. This was probably due to differences in baseline characteristics of the patients and the later completion of three loading doses in our study. Chatzirallis et al., in a prospective study comparing RNB to AFB, reported no difference in CMT change at any time point during 18-month follow-up [23]. Supportingly, both first and second year results of Protocol T revealed no difference in CMT change between AFB and RNB, irrespective of initial VA stratification. Conversely, a multicenter retrospective study based on Fight Retinal Blindness (FRB) data reported significantly better CMT outcomes with AFB both at year 1 and 3 [24,25]. Though the authors included eyes that switched between these two drugs, they did not perform comparisons between switchers and non-switchers, probably due to the low volume (19 eyes at year 1 and 96 eyes at year 3) of switchers. 

In the current study, we noted no difference in mean CMT at baseline or through 2-year follow-up between AFB and RNB. However, the RNB to AFB group had significantly thicker mean CMT at baseline and through 1-year follow-up when compared to eyes in the AFB and RNB groups. Between year 1 and year 2, eyes in the RNB to AFB group still had thicker mean CMTs, but the significant difference was no longer maintained. This could be related to significantly lower injection frequencies between year 1 and year 2 in the AFB (1.92 ± 1.5) and RNB (1.98 ± 1.8) groups when compared to the RNB to AFB (3.48 ± 1.9) group. When the year 1 and year 2 anatomical results of the current study are considered, they were significantly lower than those reported in Protocol T 1- and 2-year outcomes and FRB data 1- and 3-year outcomes. This could be related to differences between studies, such as Protocol T utilized standardized follow-ups and retreatment regimens, and FRB data included only treatment naïve eyes with more frequent visits and injections. Though there is no other study to make a head-to-head comparison with, in this comparative real-life study all of the drug groups showed substantial improvement in CMT by month 3 with further improvement through month 6. Though the RNB to AFB group started with a significantly thicker CMT and maintained this through 1 year, comparable CMT measurements were observed afterwards.

Another interesting point to highlight from our study was that all 3 groups did not show significant differences regarding BCVA improvement at any time point of the 2-year follow-up. Our results were in line with functional results at first and second year reports of Protocol T [8,9]. Another prospective comparative study reported similar visual outcomes for AFB and RNB through 18 month follow-up [23]. Conversely, the FRB 1-year report reflected greater visual gains with AFB than with RNB. However, this was no longer maintained at the 3 year mark. The proportions of eyes that gained ≥10 letters were similar (varying between 45% and 50%) across treatment groups at 1 year in Protocol T when the initial VA is good (≥69 letters). The percentage of eyes that lost ≥10 letters was also quite low and similar (1–4%). When the initial VA was worse (<69 letters), the frequency of ≥10 letters gainers (60–77%) and ≥10 letters losers (1–4%) were also similar. Supportingly, the FRB 1-year report reflected similar outcomes for AFB and RNB groups. In our study, we also found similar frequencies of ≥10 letters gainers (38–43%) and ≥10 letters losers (9–13%) across treatment groups at 1 year. At year 2, protocol T revealed a similar proportion of eyes gained and lost ≥10 letters across treatment groups when stratified according to baseline vision, except for the increased frequency of losing ≥10 letters in eyes with good vision at baseline when compared to 1 year outcomes. Interestingly, the FRB 3-year report reflected worse outcomes with a frequency of around 30% for ≥10 letters gainers and a frequency of 10% for ≥10 letters losers in AFB and RNB groups. This could be due to it including data for eyes that did not complete the prespecified follow-up of 36 months and for eyes that switched between AFB and RNB. We also found similar but slightly worse outcomes (around 40% for ≥10 letters gainers and around 15% for ≥10 letters losers) at year 2 when compared to year 1. Unsurprisingly, visual improvement in our real-world study and the FRB data was lower than the visual improvement in pivotal clinical trials, such as Protocol T.

Apart from comparing AFB to RNB, we conducted this study to discover if switching between anti-VEGFs in poor responding eyes differs from sustaining treatment with the same agent in the long-term. We designated the switch arm of the study as eyes switched from RNB to AFB since the opposite is quite rare in routine clinical practice [24]. A recent systematic review of papers reported outcomes of the RNB to AFB switch showed strong evidence for a significant reduction of macular edema, however, the evidence for significant visual improvements after the RNB to AFB switch was weak [26]. The sample size was quite low, varying between 20 and 50 eyes in each study, making a total of 188 eyes [27,28,29,30]. The mean number of injections before the switch varied widely between 5 and 21 injections. Contrary to this, our study alone is one of the largest studies, as it included 141 eyes in the RNB to AFB switch group. The mean number of injections prior to the switch was around 6 injections in the current study, which is in line with routine clinical practice as the switch is mostly performed after 3–6 injections. Inspired by the findings from protocol I indicating that the chronic persistence of edema does not preclude the eyes from obtaining favorable visual outcomes at 3 years, Bressler et al. performed a post hoc analysis of Protocol T to observe the effects of persistent edemas detected at 6 months on 2-year visual outcomes [31]. They found similar visual improvements with similar frequencies of injections and additional treatments (e.g., focal/grid laser) in eyes with and without chronic persistent edema across treatment arms, irrespective of stratification by initial VA. Based on their findings, they concluded: “Therefore, caution should be exercised when considering switching therapies for DME if there is a limited response following 3 or more initial anti-VEGF injections”. Supporting this notion, eyes that underwent the RNB to AFB switch showed slightly inferior but not significantly worse visual outcomes at both year 1 and year 2 when compared to non-switchers (AFB or RNB only) in the current study. Comparatively, the AFB group needed significantly fewer injections in the first year and fewer visits in the second year, while the RNB to AFB group needed significantly more injections in the second year.

Through the 2 years of the current study, intravitreal dexamethasone implant injection was required less frequently in the AFB group than the RNB and RNB to AFB switch groups. Similarly, the number of macular lasers (focal/grid/subthreshold) was also lower in the AFB group in the current study. While dexamethasone implant injection was not allowed as per the study protocol, the Protocol T 2-year report revealed lower frequencies of macular laser in the AFB group than the RNB and BVZ groups. However, the FRB 1- and 2-year reports reflected no differences in the need for steroid injection or macular laser between AFB and RNB groups even after eyes were stratified by initial VA.

Similar to the Protocol T 2-year report, elevation of intraocular pressure was the most frequent ocular AE in the current study. Differently, the FRB 3-year report presented preretinal vitreous hemorrhage as the most frequent AE. We did not see any cases of endophthalmitis or sterile inflammation through 2-year follow-up. Supporting the Protocol T data, rates of systemic AE were also quite low and similar across study groups.

Multicenter real-world studies are complementary to RCTs to provide further evidence for obtaining the best outcomes in patients with DME. We know there are several limitations for retrospective studies. Firstly, there was a lack of prospective randomization of drug allocation. Treatment decision, dosing frequency, and the timing of treatment switch may vary among retina specialists performing at different centers. Secondly, we also did not look at OCT characteristics in detail (e.g., ellipsoid zone disruption) at the beginning, which may affect visual prognosis in the long-term. Additionally, there was no subgroup analysis according to baseline VA stratification. On the other hand, the strengths of the current study include multiple comparisons between non-switchers and switchers, which has not been performed to date in such a large sample size.

In conclusion, this multicenter retrospective study showed similar visual improvements in non-switchers (AFB and RNB patients) and switchers (RNB to AFB) through 2-year follow-up, however, AFB patients required fewer injections, center visits, and additional treatments. Though the proportions of eyes showing good responses (≥20% thinning of CMT) were similar among three groups at month 3 and month 6, the RNB to AFB group had thicker mean CMTs through 12 months and caught up with the other two groups after a higher number of injections. Moreover, patients in all groups did not exhibit a high risk for ocular or systemic AEs.

## Figures and Tables

**Table 1:** Baseline characteristics of the study groups.

	Aflibercept (*n* = 212)	Ranibizumab (*n* = 461)	Ranibizumab-Aflibercept Switch (*n* = 141)	*p* Value
Age (years: mean ± SD)	60.78 ± 8.3	61.07 ± 8.7	60.45 ± 8.8	0.74
Gender (%) Male Female	54.8 45.2	50.4 49.6	65.6 34.4	0.002 *
Type of DM (%) Type 1 Type 2	7.2 92.8	8.7 91.3	12.4 87.6	0.074
DM duration (years: mean ± SD)	14.77 ± 6.4	15.11 ± 6.2	14.27 ± 6.2	0.396
Treatment of DM (%) Insulin Insulin + OAD OAD	54.9 24.1 21	52.1 19.1 28.8	53.7 21.5 24.8	0.349
Laterality (%) Right Eye Left Eye	51.6 48.4	53 47	54.4 45.6	
DME treatment status (%) Naïve Non-naïve	62.7 37.3	62.1 37.9	56.4 43.6	0.4
Prior DME treatments (*n*) Bevacizumab Focal, grid laser Intravitreal triamsinolone Subthreshold laser Dual treatment Triple treatment	34 25 3 3 18 0	51 78 9 4 40 6	19 25 1 1 16 3	0.331
Prior PRP treatment (%) Yes No	33.4 66.6	26.6 73.4	29.5 70.5	<0.01 *
Lens status (%) Phakic Pseudophakic	72.8 27.2	77.5 22.5	70.4 29.6	0.063
DR grade (%) NPDR PDR	71.5 28.5	70.3 29.7	79.5 20.5	0.339
Comorbidities (*n*) Hypertension Coronary heart disease Hyperlipidemia Nephropathy Cerebrovascular accident	79 35 25 10 1	167 32 65 16 3	60 15 12 5 0	0.02 *

DM: diabetes mellitus, DME: diabetic macular edema, DR: diabetic retinopathy, *n*: number, NPDR: nonproliferative diabetic retinopathy, OAD: oral antidiabetic drug, PDR: proliferative diabetic retinopathy, PRP: panretinal photocoagulation, SD: standard deviation, * statistically significant (*p* < 0.05).

**Table 2:** Clinical characteristics of the study groups during 2-year follow-up.

	Aflibercept (*n* = 212)	Ranibizumab (*n* = 461)	Ranibizumab-Aflibercept Switch (*n* = 141)	*p* Value
Three loading doses (%) Yes No	55.8 44.2	54 46	63.8 36.2	<0.01 *
Loading dose completion (weeks: mean + SD)	14.29 ± 2.9	13.79 ± 3.3	13.30 ± 2.4	0.091
Anti-VEGF switch (injections: mean ± SD)	-	-	5.82 ± 2.2 (3–10)	-
Number of visits 1st year 2nd year	7.57 ± 1.9 4.12 + 1.9	7.68 ± 2 6.28 + 2.1	7.76 ± 2.1 6.8 + 2.1	0.55 <0.01 *
Number of injections 1st year 2nd year	4.43 ± 1.7 1.92 ± 1.5	5.3 ± 1.8 1.98 ± 1.8	5.39 ± 1.8 3.48 + 1.9	<0.01 * <0.01 *
Additional treatments (%) Dexamethasone implant Focal/Grid/Subthreshold laser Vitrectomy PRP	11.3 17.4 0.9 9.2	15.7 25.3 1.2 12.2	17.4 32.1 0 6.7	<0.01 * <0.01 *
Ocular side effects (*n*) IOP increase Glaucoma Vitreous hemorrhage	2 1 1	5 2 2	1 1 1	
Systemic side effects (*n*) Myocardial infarction Cerebrovascular accident	1 1	2 1	3	

IOP: intraocular pressure, *n*: number, PRP: panretinal photocoagulation, SD: standard deviation, VEGF: vascular endothelial growth factor, * statistically significant (*p* < 0.05).

**Table 3:** Best corrected visual acuity (BCVA) and central macular thickness (CMT) changes through 2-year follow-up among the study groups.

	Aflibercept (*n* = 212)	Ranibizumab (*n* = 461)	Ranibizumab-Aflibercept Switch (*n* = 141)	*p* Value
BCVA (logMAR: mean ± SD)				
Baseline	0.62 ± 0.38	0.61 ± 0.36	0.61 ± 0.38	0.83
3rd month	0.49 ± 0.35	0.46 ± 0.35	0.50 ± 0.36	0.35
6th month	0.44 ± 0.34	0.50 ± 0.38	0.50 ± 0.37	0.049
9th month	0.45 ± 0.36	0.49 ± 0.35	0.51 ± 0.40	0.16
12th month	0.44 ± 0.37	0.46 ± 0.37	0.47 ± 0.38	0.72
15th month	0.46 ± 0.37	0.48 ± 0.38	0.50 ± 0.34	0.65
18th month	0.45 ± 0.37	0.47 ± 0.38	0.53 ± 0.38	0.46
21st month	0.47 ± 0.38	0.50 ± 0.36	0.54 ± 0.37	0.5
24th month	0.46 ± 0.38	0.50 ± 0.37	0.53 ± 0.36	0.46
CMT (µm: mean ± SD)				
Baseline	450.5 ± 122.6	442.0 ± 116.0	479.0 ± 129.6	<0.01 *
3rd month	338.0 ± 107.5	347.0 ± 113.3	381.9 ± 124.5	<0.01 *
6th month	341.4 ± 104.0	346.7 ± 109.2	395.3 ± 135.7	<0.01 *
9th month	333.4 ± 118.5	352.4 ± 122.4	388.7 ± 130.7	<0.01 *
12th month	333.0 ± 95.20	342.0 ± 119.1	369.2 ± 123.7	<0.01 *
15th month	354.8 ± 116.2	359.8 ± 137.4	368.3 ± 130.6	0.11
18th month	354.7 ± 126.8	356.0 ± 122.6	362.1 ± 123.5	0.45
21st month	368.8 ± 124.8	371.1 ± 154.9	372.0 ± 129.7	0.39
24th month	366.7 ± 112.1	369.8 ± 104.6	373.4 ± 163.1	0.27

BCVA: best corrected visual acuity, CMT: central macular thickness, µm: micrometer, * statistically significant (*p* < 0.05).

## Data Availability

The datasets generated during and/or analyzed during the current study are available from the corresponding author on reasonable request.

## References

[1] IDF (2021). Diabetes Atlas.

[2] Thomas R., Halim S., Gurudas S., Sivaprasad S., Owens D. (2019). IDF Diabetes Atlas: A review of studies utilising retinal photography on the global prevalence of diabetes related retinopathy between 2015 and 2018. Diabetes Res. Clin. Pr..

[3] Strain W., Cos X., Hirst M., Vencio S., Mohan V., Vokó Z., Yabe D., Blüher M., Paldánius P. (2014). Time to do more: Addressing clinical inertia in the management of type 2 diabetes mellitus. Diabetes Res. Clin. Pr..

[4] Bressler S.B., Glassman A.R., Almukhtar T., Bressler N.M., Ferris F.L., Googe J.M., Gupta S.K., Jampol L.M., Melia M., Wells J.A. (2016). Five-Year Outcomes of Ranibizumab With Prompt or Deferred Laser Versus Laser or Triamcinolone Plus Deferred Ranibizumab for Diabetic Macular Edema. Am. J. Ophthalmol..

[5] Heier J.S., Korobelnik J.-F., Brown D.M., Schmidt-Erfurth U., Do D.V., Midena E., Boyer D.S., Terasaki H., Kaiser P.K., Marcus D.M. (2016). IIntravitreal aflibercept for diabetic macular edema: 148-Week results from the VISTA and VIVID studies. Ophthalmology.

[6] Boyer D.S., Nguyen Q.D., Brown D.M., Basu K., Ehrlich J.S. (2015). Outcomes with as-needed ranibizumab after initial monthly therapy: Long-term Outcomes of the Phase III RIDE and RISE trials. Ophthalmology.

[7] Schmidt-Erfurth U., Lang G.E., Holz F.G., Schlingemann R.O., Lanzetta P., Massin P., Gerstner O., Bouazza A.S., Shen H., Osborne A. (2014). Three-year outcomes of individualized ranibizumab treatment in patients with diabetic macular edema: The RESTORE extension study. Ophthalmology.

[8] Wells J.A., Glassman A.R., Ayala A.R., Jampol L.M., Bressler N.M., Bressler S.B., Brucker A.J., Ferris F.L., Hampton G.R., Jhaveri C. (2016). Aflibercept, bevacizumab, or ranibizumab for diabetic macular edema: Two-year results from a comparative effectiveness randomized clinical trial. Ophthalmology.

[9] Wells J.A., Glassman A.R., Ayala A.R., Jampol L.M., Aiello L.P., Antoszyk A.N., Arnold-Bush B., Baker W.C., Bressler N.M., Diabetic Retinopathy Clinical Research Network (2015). Aflibercept, bevacizumab, or ranibizumab for diabetic macular edema. N. Engl. J. Med..

[10] Elman M.J., Aiello L.P., Beck R.W., Bressler N.M., Bressler S.B., Edwards A.R., Ferris F.L., Friedman S.M., Glassman A.R., Diabetic Retinopathy Clinical Research Network (2010). Randomized Trial Evaluating Ranibizumab Plus Prompt or Deferred Laser or Triamcinolone Plus Prompt Laser for Diabetic Macular Edema. Ophthalmology.

[11] Nguyen Q.D., Brown D.M., Marcus D.M., Boyer D.S., Patel S., Feiner L., Gibson A., Sy J., Rundle A.C., Hopkins J.J. (2012). Ranibizumab for Diabetic Macular Edema: Results from 2 phase III randomized trials: RISE and RIDE. Ophthalmology.

[12] Bahrami B., Hong T., Schlub T.E., Chang A.A. (2019). Aflibercept for persistent diabetic macular edema: Forty-eight-week outcomes. Retina.

[13] Chen Y.-Y., Chang P.-Y., Wang J.-K. (2017). Intravitreal Aflibercept for Patients With Diabetic Macular Edema Refractory to Bevacizumab or Ranibizumab: Analysis of Response to Aflibercept. Asia-Pac. J. Ophthalmol..

[14] Liu Y., Cheng J., Gao Y., Qin L., Min X., Zhang M. (2020). Efficacy of switching therapy to aflibercept for patients with persistent diabetic macular edema: A systematic review and meta-analysis. Ann. Transl. Med..

[15] Bressler S.B., Qin H., Beck R.W., Chalam K.V., Kim J.E., Melia M., Wells J.A. (2012). Factors Associated With Changes in Visual Acuity and Central Subfield Thickness at 1 Year After Treatment for Diabetic Macular Edema With Ranibizumab. Arch. Ophthalmol..

[16] Pieramici D.J., Wang P.-W., Ding B., Gune S. (2016). Visual and Anatomic Outcomes in Patients with Diabetic Macular Edema with Limited Initial Anatomic Response to Ranibizumab in RIDE and RISE. Ophthalmology.

[17] Sivaprasad S., Crosby-Nwaobi R., Heng L.Z., Peto T., Michaelides M., Hykin P. (2013). Injection frequency and response to bevacizumab monotherapy for diabetic macular oedema (BOLT Report 5). Br. J. Ophthalmol..

[18] Sorour O., Liu K., Mehta N., Braun P., Gendelman I., Nassar E., Baumal C.R., Witkin A.J., Duker J.S., Waheed N.K. (2020). Visual and anatomic outcomes of sustained single agent anti-VEGF treatment versus double anti-VEGF switching in the treatment of persistent diabetic macular edema. Int. J. Retin. Vitr..

[19] Demircan A., Alkin Z., Yesilkaya C., Demir G., Kemer B. (2018). Comparison of Intravitreal Aflibercept and Ranibizumab following Initial Treatment with Ranibizumab in Persistent Diabetic Macular Edema. J. Ophthalmol..

[20] Gregori N.Z., Feuer W., Rosenfeld P.J. (2010). Novel method for analyzing snellen visual acuity measurements. Retina.

[21] Sun J.K., Josic K., Melia M., Glassman A.R., Bailey C., Chalam K.V., Chew E.Y., Cukras C., Grover S., Jaffe G.J. (2021). Conversion of Central Subfield Thickness Measurements of Diabetic Macular Edema Across Cirrus and Spectralis Optical Coherence Tomography Instruments. Transl. Vis. Sci. Technol..

[22] Dugel P.U., Campbell J.H., Kiss S., Loewenstein A., Shih V., Xu X., Holekamp N.M., Augustin A.J., Ho A.C., Gonzalez V.H. (2019). Association Between Early Anatomic Response To Anti-Vascular Endothelial Growth Factor Therapy And Long-Term Outcome In Diabetic Macular Edema: An Independent Analysis of Protocol i Study Data. Retina.

[23] Chatzirallis A., Theodossiadis P., Droutsas K., Koutsandrea C., Ladas I., Moschos M.M. (2020). Ranibizumab versus aflibercept for diabetic macular edema: 18-month results of a comparative, prospective, randomized study and multivariate analysis of visual outcome predictors. Cutan. Ocul. Toxicol..

[24] Bhandari S., Nguyen V., Fraser-Bell S., Mehta H., Viola F., Baudin F., Gabrielle P.-H., Creuzot-Garcher C., Gillies M., Barthelmes D. (2020). Ranibizumab or Aflibercept for Diabetic Macular Edema: Comparison of 1-Year Outcomes from the Fight Retinal Blindness! Registry. Ophthalmology.

[25] Gabrielle P.-H., Nguyen V., Creuzot-Garcher C., Arnold J.J.M., Mehta H., Duran M.A., Bougamha W.M., Carreño E., Viola F., Squirrell D.F. (2020). Three-Year Treatment Outcomes Of Aflibercept Versus Ranibizumab For Diabetic Macular Edema: Data from the Fight Retinal Blindness! Registry. Retina.

[26] Madjedi K., Pereira A., Ballios B.G., Arjmand P., Kertes P.J., Brent M., Yan P. (2022). Switching between anti-VEGF agents in the management of refractory diabetic macular edema: A systematic review. Surv. Ophthalmol..

[27] Egan C., Zhu H., Lee A., Sim D., Mitry D., Bailey C., Johnston R., Chakravarthy U., Denniston A., Tufail A. (2017). The United Kingdom Diabetic Retinopathy Electronic Medical Record Users Group, Report 1: Baseline characteristics and visual acuity outcomes in eyes treated with intravitreal injections of ranibizumab for diabetic macular oedema. Br. J. Ophthalmol..

[28] Ehlers J.P., Wang K., Singh R.P., Babiuch A.S., Schachat A.P., Yuan A., Reese J.L., Stiegel L., Srivastava S.K. (2018). A prospective randomized comparative dosing trial of ranibizumab in bevacizumab-resistant diabetic macular edema: The REACT study. Ophthalmol. Retin..

[29] Ehrlich R., Pokroy R., Segal O., Goldstein M., Pollack A., Hanhart J., Barak Y., Kehat R., Shulman S., Vidne O. (2019). Diabetic macular edema treated with ranibizumab following bevacizumab failure in Israel (DERBI study). Eur. J. Ophthalmol..

[30] Fechter C., Frazier H., Marcus W.B., Farooq A., Singh H., Marcus D.M. (2016). Ranibizumab 0.3 mg for Persistent Diabetic Macular Edema After Recent, Frequent, and Chronic Bevacizumab: The ROTATE Trial. Ophthalmic Surg. Lasers Imaging Retin..

[31] Bressler N.M., Beaulieu W., Glassman A.R., Blinder K.J., Bressler S.B., Jampol L.M., Melia M., Wells J.A., Diabetic Retinopathy Clinical Research Network (2018). Persistent Macular Thickening Following Intravitreous Aflibercept, Bevacizumab, or Ranibizumab for Central-Involved Diabetic Macular Edema With Vision Impairment: A Secondary Analysis of a Randomized Clinical Trial. JAMA Ophthalmol.

